# Uncovering the Role of Oxidative Imbalance in the Development and Progression of Bronchial Asthma

**DOI:** 10.1155/2021/6692110

**Published:** 2021-03-03

**Authors:** Luiz H. C. Vasconcelos, Sarah R. D. Ferreira, Maria da C. C. Silva, Paula B. Ferreira, Iara L. L. de Souza, Fabiana de A. Cavalcante, Bagnólia A. da Silva

**Affiliations:** ^1^Laboratório de Farmacologia Funcional Prof. George Thomas, Instituto de Pesquisa em Fármacos e Medicamentos, Universidade Federal da Paraíba, João Pessoa, PB 58.051-900, Brazil; ^2^Departamento de Fisiologia e Patologia, Centro de Ciências da Saúde, Universidade Federal da Paraíba, João Pessoa, PB 58.051-900, Brazil; ^3^Programa de Pós-graduação em Produtos Naturais e Sintéticos Bioativos, Centro de Ciências da Saúde, Universidade Federal da Paraíba, João Pessoa, PB 58.051-900, Brazil; ^4^Departamento de Ciências Biológicas e Saúde, Universidade Estadual de Roraima, Boa Vista, RR 69306-530, Brazil; ^5^Departamento de Ciências Farmacêuticas, Centro de Ciências da Saúde, Universidade Federal da Paraíba, João Pessoa, PB 58.051-900, Brazil

## Abstract

Asthma is a chronic inflammatory disease of the airways related to epithelial damage, bronchial hyperresponsiveness to contractile agents, tissue remodeling, and luminal narrowing. Currently, there are many data about the pathophysiology of asthma; however, a new aspect has emerged related to the influence of reactive oxygen and nitrogen species (ROS and RNS) on the origin of this disease. Several studies have shown that an imbalance between the production of ROS and RNS and the antioxidant enzymatic and nonenzymatic systems plays an important role in the pathogenesis of this disease. Considering this aspect, this study is aimed at gathering data from the scientific literature on the role of oxidative distress in the development of inflammatory airway and lung diseases, especially bronchial asthma. For that, articles related to these themes were selected from scientific databases, including human and animal studies. The main findings of this work showed that the respiratory system works as a highly propitious place for the formation of ROS and RNS, especially superoxide anion, hydrogen peroxide, and peroxynitrite, and the epithelial damage is reflected in an important loss of antioxidant defenses that, in turn, culminates in an imbalance and formation of inflammatory and contractile mediators, such as isoprostanes, changes in the activity of protein kinases, and activation of cell proliferation signalling pathways, such as the MAP kinase pathway. Thus, the oxidative imbalance appears as a promising path for future investigations as a therapeutic target for the treatment of asthmatic patients, especially those resistant to currently available therapies.

## 1. Introduction

Asthma is a chronic inflammatory disease of the airways in which many cells of the innate and adaptive immune system act together with epithelial cells to cause bronchial hyperreactivity, overproduction of mucus, remodeling of the wall, and narrowing of the airways. In this inflammatory context, a series of reactive species are produced, leading to changes in the function of the epithelium, smooth muscle cells, and the immune system and in the structure of the airway wall, leading to the pulmonary limitations that characterize this disease [1].

The oxidative stress resulting from an imbalance between oxidants, such as reactive oxygen and nitrogen species (ROS and RNS), and antioxidants, in favor of oxidants, has been called oxidative distress, a condition known to lead to biological damage. ROS refer to radicals derived from O_2_ metabolism, as well as to nonradical derivatives reactive to O_2_ (for example, hydrogen peroxide), and the term RNS refer to radicals of nitrogen reactive to other molecules in which the reactive center is nitrogen [2].

The most common ROS and RNS, in the order of reactive capacity, are as follows: superoxide anion (O_2_^-•^), hydrogen peroxide (H_2_O_2_), hydroxyl radical (HO^•^), singlet oxygen (1O_2_), peroxyl radical (HO_2_^•^), nitric oxide (^•^NO), peroxynitrite (ONOO^−^), perhydroxy radical (HO_2_^•^), hydroperoxyl radical (ROOH^•^), hypochlorous acid (HClO), ozone (O_3_), and nitric dioxide (NO_2_) [3].

Although the generation of oxidative molecules is part of normal metabolism, the intracellular levels of ROS and RNS are kept at low levels of concentrations under normal physiological conditions, acting as important mediators involved in the regulation of some cellular processes, such as growth, adhesion, cell differentiation and death, modulation of gene expression, and signalling of cellular transduction pathways [4, 5].

The involvement of reactive species in the regulation of intracellular signalling occurs through posttranslational modification of proteins sensitive to redox alteration, including several receptor and ion channels, kinases and phosphatases, caspases, and transcription factors, which have functionally significant cysteine residues, liable of undergoing oxidation [6]. Thus, ROS can oxidize cysteine sulfhydryl groups (Cys-SH) with the formation of sulfenic (Cys-SOH) and then sulfinic (Cys-SO_2_H) and sulfonic acids (Cys-SO_3_H), resulting in the alteration of activity and functioning of the protein participating in signal transduction pathways [7].

The lungs are continuously exposed to a variety of oxidants that differ in type and degree, and can easily overcome the resulting oxidative stress with the help of an enzymatic and nonenzymatic antioxidant network. The main antioxidant enzymes include superoxide dismutase (SOD), catalase (CAT), and glutathione-dependent enzymes, such as glutathione peroxidase (GSH-Px), glutathione S-transferase (GST), glutathione reductase (GSH), and glutathione synthetase [8].

While airway inflammation and asthma tend to increase the production of ROS and RNS, mainly through the activity of eosinophils and neutrophils [9], accumulated evidences suggest the participation of oxidative stress in asthma genesis and modulation [10, 11].

Thus, this work is aimed at exploring the respiratory system as a place for the production of reactive species. It also explores the role of pulmonary antioxidant systems in relation to the origin of inflammatory diseases of the respiratory system, particularly bronchial asthma. In addition, it explores the mechanisms involved with these effects mediated by oxidative imbalance.

## 2. Origin of ROS in the Respiratory System and Its Antioxidant Barrier

The respiratory system is an environment that presents an increased risk of suffering from damage caused by oxidative stress due to the high susceptibility to interactions with environmental factors. The lungs are exposed to exogenous ROS, originating from pollutants and cigarette smoke, and endogenous ROS, produced diffusely as a by-product of cell metabolism [12], mainly by inflammatory cells, such as eosinophils, macrophages, and neutrophils [13], as well as by fibroblasts and epithelial cells [2, 12]).

The production of ROS in the respiratory system occurs at cellular sites, such as mitochondria, microsomes, and enzymes (such as xanthine oxidase, monooxygenase P450, cyclooxygenase, lipoxygenase, indolamine dioxygenase, and monoamine oxidase) [14, 15]. Of these, mitochondria are the major contributors to the formation of ROS; it is estimated that 1 to 3% of the electron flow in this organelle forms a superoxide anion [16].

The initial step for the formation of these free radicals is the activation of the enzyme complex of nicotinamide adenine dinucleotide reduced phosphate (NADPH) oxidase, present in the cell membrane, and the generation of the superoxide anion [17]. This compound can be spontaneously or enzymatically dismuted to H_2_O_2_ and molecular oxygen (O_2_). Both O_2_^−^ and H_2_O_2_ can react, in the presence of iron or other metals, to form the most potent OH° radical [18].

In the context of asthma, inflammatory cells are the main sources of ROS. Granulocytes contain peroxidases (such as myeloperoxidase and eosinophilic peroxidase), which catalyze the reaction of H_2_O_2_ with halides, leading to the formation of hypohalides, such as hypochlorous acid (HClO) [19]. Antigenic challenges in asthmatic patients increase the formation of ROS by eosinophils [20]. In addition, leukocytes circulating in the blood increase the production of superoxide anion, indicating that both pulmonary and intravascular inflammatory cells contribute to oxidative stress in asthma [21]. In this case, the airway epithelium of asthmatics produces few antioxidants, which explains the loss of the epithelium effectiveness in maintaining airway homeostasis [22].

To counterbalance the formation of these ROS, the respiratory system has enzymatic and nonenzymatic antioxidant systems, in order to maintain normal levels of these free radicals, necessary for the perfect cellular functioning. The major enzyme systems in the airways are the SOD complex, which converts the superoxide anion into hydrogen peroxide; catalase, which converts hydrogen peroxide into water and molecular oxygen; and glutathione peroxidase (GSH-Px) and reductase (GR), which inactivate hydrogen peroxide and other hydroperoxides [23–26]. Among these antioxidant systems, SOD is highly expressed extracellularly in the lungs, around the airways and smooth muscle, being abundant in the epithelium [27], and plays an important role in combating oxidative stress in asthma. In this context, asthmatic patients are deficient in antioxidant defenses, with the resulting burden of oxidative stress contributing to pulmonary dysfunction [28].

**Figure 1:**
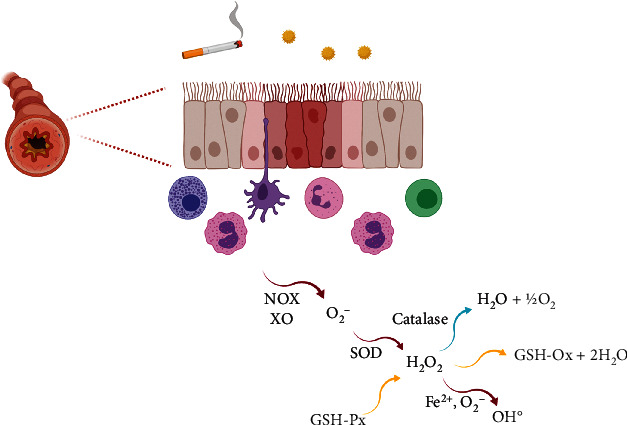
Formation of ROS by inflammatory cells and the antioxidant defenses on airways. Created with BioRender.com.

The main aspects related to the formation of reactive species in the respiratory system associated with diseases such as asthma are shown in Figure 1.

## 3. Nitric Oxide: Good Guy or Villain in the Airways?

Nitric oxide (NO) has been well described in the literature as an important signalling molecule involved in the regulation of many mammalian physiological and pathophysiological processes, particularly in the lung [29]. It plays a role in regulating both pulmonary vascular tone and bronchomotor tone in the airways. In addition, NO participates in the host's inflammation and defense against infection through changes in vascular permeability, changes in epithelial barrier function and repair, cytotoxicity, ciliary motility regulation, mucus secretion, and inflammatory cell infiltration [30]. Thus, these multiple NO functions have been implicated in the pathogenesis of chronic inflammatory diseases of the airways [31].

NO is produced by a family of nitric oxide synthases (NOS), which metabolize L-arginine through the N-hydroxy intermediate L-arginine (NOHA) to form NO and L-citrulline using molecular oxygen and NADPH as cofactor. Three NOS isoenzymes have been identified in mammals, with variable distributions. Neuronal NOS (nNOS or NOS I) and endothelial NOS (eNOS or NOS III) are the constitutively expressed forms in the airway epithelium, nonadrenergic noncholinergic neurons (iNANC) and in endothelial cells of the airway vessels. Its activity is regulated by intracellular calcium, with rapid onset of activity and production of small amounts of NO in the order of magnitude of picomoles, that play an important role in maintaining respiratory homeostasis. Otherwise, the inducible NOS (iNOS or NOS II) is transcriptionally regulated by proinflammatory stimuli, with the ability to produce large amounts (nanomolar concentrations) of NO for a long period of time [30, 32]. About iNOS, its positive regulation is observed in the lungs of asthmatics, and the increased levels of exhaled NO are well described in patients with asthma, being a marker of the severity of the disease [33].

In the early phase of the asthmatic response, NO plays an important role in reducing airway hyperresponsiveness (AHR), and the hyperreactive response is related to NO deficiency, either due to the greater activity of the enzyme arginase, which prevents the conversion to NO and L-citrulline by NOS, or due to the lower activity of NOS constitutive isoforms. In this sense, the decrease in eNOS or nNOS expression has already been observed in guinea pigs exposed to repeated allergenic challenge and in asthmatic patients, respectively [34, 35]. In contrast, in the late phase of the asthmatic response, the increased activity of iNOS, which produces large amounts of NO, is responsible for AHR [36, 37].

This increased activity (or increased expression) of iNOS occurs due to the action of inflammatory cytokines [29, 33]. Corroborating these propositions, it was observed in models of acute asthma in guinea pigs that, in the rapid phase of the asthmatic response, the levels of exhaled NO decrease, and the iNOS expression is not altered as well; this is in contrast to the late phase, in which exhaled NO levels are high, as well as iNOS expression [37–39].

Inflammation and AHR in asthma are not the result of increased production of NO itself, but are due to the formation of the free radical, strong oxidizing agent, peroxynitrite, resulting from the reaction of NO with the superoxide anion in the airways [40, 41]. Peroxynitrite activates eosinophils, increases mucin 5AC (MUC5AC) expression, increases microvascular permeability, induces epithelial damage, and increases contraction of smooth muscle in the airways [42–44]. In this context, studies have shown that airway epithelial cells and bronchial biopsy inflammatory cells from asthmatic patients, as well as from sensitized guinea pigs, show an increase in nitrotyrosine labeling (a marker of tyrosine residue nitrosylation in proteins), which is also correlated with the increase of exhaled NO, expression of iNOS and AHR, and eosinophilic inflammation, having as an intermediary the action of free radicals and oxidative stress [41, 43, 45].

Recent studies have described a new source of superoxide anion, the decoupled endothelial nitric oxide (eNOS) synthase, a process that occurs when eNOS is not associated with cofactor or substrate [46]. This process can occur due to the oxidative action of peroxynitrite on the tetrahydrobiopterine cofactor [47] or by a reduction in the availability of L-arginine, common in asthma due to the increased expression of the enzyme arginase [31].

**Figure 2:**
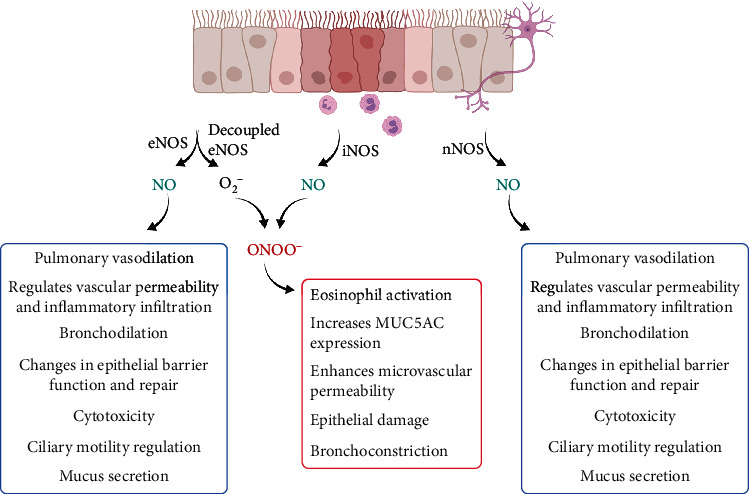
Summarized schema of the multiple NO functions in the airways. Created with BioRender.com.

A summary of the multiple NO functions in the airways are presented in Figure 2.

The formation of the peroxynitrite radical, by itself, already produces harmful effects on the respiratory system that contribute to inflammatory damage, as well as reducing the availability of NO that has a role in regulating airway smooth muscle tone. A key molecule in increasing oxidative stress and decreasing NO availability is asymmetric dimethylarginine (ADMA). It is a product of posttranslational methylation of L-arginine, synthesized by the protein arginine methyltransferase (PRMT) and degraded by dimethylamino hydrolase (DDAH). ADMA binds to different NOS isoforms, inhibiting them and competing with NO for their binding site. Asthmatic subjects have high levels of ADMA, which may be one of the factors responsible for the asthmatic worsening [47].

## 4. Assessment of Oxidative Stress in the Respiratory System

The increase in ROS production enhances lipid peroxidation, as well as damage to proteins, aggravating airway inflammation through multiple mechanisms, including the release of inflammatory mediators and effects on mucus production and smooth muscle [48]. On the other hand, impaired antioxidant defense mechanisms contribute to oxidative damage in asthma [49]. Among the markers of oxidative stress are those specific to the lungs [50–53]. In this context, while increased levels of these markers in the plasma indicate systemic oxidative damage, which may or may not have originated from the respiratory system, the measurement of these markers in lung samples more accurately reflects the oxidative stress that occurs in the respiratory system [54].

The markers of pulmonary oxidative stress, determined in rodents and human subjects, are aldehyde products from the oxidative decomposition of polyunsaturated fatty acids in the membrane, such as malondialdehyde (MDA), hexanal, heptanal, nonanal, acrolein, 4-hydroxyhexanal (4-HHE), and 4-hydroxinonenal (4-HNE) [55], in addition to isoprostane, a product of the peroxidation of arachidonic acid by ROS, responsible for producing prostanoids in a pathway independent of cyclooxygenase, for example the 8-iso-PGF_2*α*_ [56–58].

In this context, an increase in MDA levels in a model of chronic allergic asthma in mice (an indicator of elevated oxidative damage [59, 60]) and the rise in MDA levels have also been reported in other studies as a characteristic of oxidative damage caused by asthma, both in mice and rats [61–63]. Additionally, the increased levels of 8-iso-PGF_2*α*_ have been described in a guinea pig model of chronic allergic lung inflammation [64]. Furthermore, Pinkerton et al. [65] evidenced increased levels of 3-nitrotyrosine and 8-isoprostane markers in a murine model of *Chlamydia*-induced steroid-resistant asthma.

## 5. Actions of Reactive Species in the Respiratory System

Several studies have already reported the effects of reactive species on the functioning of airway smooth muscle, involving numerous cells and signalling pathways being activated and/or inhibited by it. These effects are related to exacerbated production of ROS and RNS caused by the chronic inflammatory condition [66, 67], deficiency of intrinsic (for example, glutathione) or extrinsic (for example, natural vitamins and antioxidants in the diet) antioxidants [68–70], decreased activity or dysfunction of antioxidant enzymes [71], or excessive activity of prooxidative enzymes [72].

Hydrogen peroxide and increased oxygen levels have been shown to induce contraction of the guinea pig trachea [73] and to stimulate MAPKs involved in regulating the proliferation of tracheal myocytes [74]. In addition to its direct effects, studies show that ROS also influences airway reactivity to contractile and relaxing agonists; among them, the increased contractile response to acetylcholine and methacholine [75], histamine [42], serotonin [76], and the substance P [77], and the decrease in the number and function of *β*_2_ adrenergic receptors can be cited [78, 79].

Isoprostanes, such as 8-iso-PGF_2*α*_ [80], are considered markers of oxidative stress in asthmatic patients and can induce contraction of airway smooth muscle [58, 59]. In this sense, the participation of 8-iso-PGF2*α* in the increase of airway resistance associated with oxidative stress has already been evidenced in previous studies [81, 82].

In an ovalbumin-induced asthma model, Vasconcelos et al. [64] demonstrated that the airway smooth muscle hyperreactivity was due to an increase in the formation of superoxide anion and hydrogen peroxide, in addition to a greater expression of iNOS, which culminated in a greater production of peroxynitrite radical and, as a consequence, 8-iso-PGF_2*α*_. In addition, it was shown that the antioxidant defenses in the asthmatic animals were impaired, contributing to oxidative stress.

Another marker of oxidative stress is 3-nitrotyrosine (3-NT), which is formed from the nitration of the amino acid tyrosine and has already been found in the airway epithelium, lung parenchyma, and inflammatory cells of asthmatic individuals at high levels [41, 83]. Tyrosine nitration can inhibit protein kinase phosphorylation, thereby interfering with the signal transduction mechanism [84], in addition to this role in increasing eosinophil chemotaxis by RANTES and IL-5 [85].

*In vitro* studies also demonstrated that the nitration of a specific tyrosine residue inactivated superoxide dismutase [86, 87], causing a reduction in its activity and generating an increase in the concentration of ROS and RNS, with consequent tissue damage [88]. Sugiura and Ichinose [89] also described a positive correlation between 3-nitrotyrosine, iNOS, and xanthine oxidase (XO) activity, indicating that together, iNOS and XO may be associated with the generation of RNS in the airways of patients with asthma.

The targets of ROS include catalytic receptors, phosphatases, and phospholipids, in addition to proteins from the MAPK pathway [90]. ROS comprise a type of damage-associated molecular pattern molecules (DAMP), which can activate dendritic cells, stimulating nuclear factor-*κ*B (NF-*κ*B) and triggering the inflammatory response [91]. In addition, the production of ROS by NADPH oxidase is related to the proliferation of airway smooth muscle in response to growth factors, followed by the activation of NF-*κ*B, culminating in tissue remodeling and narrowing of the air lumen [92].

Neutrophils also play an important role in the production of ROS. Stimulated by various respiratory inflammatory diseases, such as asthma and chronic obstructive pulmonary disease (COPD), after activation, they release proinflammatory ROS and serine proteases, including neutrophil elastase and proteinase-3. These proteins degrade pulmonary elastin fibers, induce mucus production, and stimulate the secretion of metalloproteinase (MMP) subtypes 8 or 9 that break down elastin and collagen, worsening respiratory symptoms. Through positive feedback, neutrophils themselves also release factors, such as leukotriene B_4_ (LTB4) acting as a bronchoconstrictor, and IL-8, in which both attract other neutrophils and perpetuate chronic inflammation [93, 94].

Another important cellular signalling covers the activity of the nuclear factor erythroid 2-related factor (Nrf2), which is a transcription factor that regulates the expression of genes involved in the protection against oxidative damage [95, 96]. Physiologically, Nrf2 is bound and inhibited by Kelch-like ECH-associated protein 1 (Keap1), which prevents its binding to the antioxidant response element and is continuously degraded by the proteasome pathway. However, in the presence of ROS, increased during asthma, Keap1 inhibition occurs and Nrf2 migrates to the nucleus, increasing the synthesis of antioxidant enzymes that include GSH- and thyrodoxin- (TXN-) dependent systems [97, 98].

Changes and increased activity of Nrf2 are associated with several respiratory diseases. In asthma, there has been an increase in susceptibility, severity, and inflammation caused by it in mice [99], as well as an increase in the proliferation of smooth muscle cells in the airways in case of alteration [100]. Similarly, in COPD, a reduction in Nrf2 expression in pulmonary macrophages is reported [101], as well as a relationship between genetic alterations of this protein and the induction of disease onset, as well as emphysema [102].

Furthermore, activation of toll-like receptors (TLRs) was also reported in pulmonary inflammation in mice [103]. These receptors are expressed in several cells in the airways, including epithelial cells, macrophages, mast cells, and dendritic cells (DCs). The stimulation of TLRs by infectious agents activates antigen-presenting cells (APC) and controls the differentiation of T helper immune cells (Th1, Th2, and Th17) in a context-dependent manner, together with the activation of mast cells for the production of cytokines [104], which increase the production of ROS. In addition to the inflammatory component, bronchial hyperresponsiveness has also been associated with activity especially of TLR2 and TLR4 [105].

A summary of the mechanisms of cell damage in the respiratory system by reactive species is presented in Table 1.

## 6. Conclusions and Perspectives

Oxidative stress is a fundamental physiological cellular process related to several adaptations; however, when deregulated, it can lead to exacerbated cellular damage, culminating in the development of chronic diseases. The respiratory system is an environment susceptible to the action of reactive species by its close contact with air particles, allergens, pollutants, and pathogens capable of triggering inflammatory diseases, such as asthma. In this sense, the compiled data collected demonstrated a possible key role of ROS and RNS in the genesis of allergic inflammatory diseases of the respiratory system, especially the superoxide anion, hydrogen peroxide, and peroxynitrite, and how altered epithelial and pulmonary antioxidant systems play an important role in this process. However, additional studies, especially in humans, are necessary in order to make it possible to extrapolate these results obtained from animal models for asthmatic patients.

It has been shown that adult asthma is associated with a low dietary intake of fruits rich in antioxidant nutrients and low plasma vitamin C levels, suggesting that diet may be a potentially modifiable risk factor for the development of asthma [106]. The use of thiol compounds such as antioxidants in antiasthmatic therapy should be explored, due to the possible ability to mimic the endogenous effects promoted by GSH, as well as the use of plant-derived polyphenolic compounds, such as flavonoids. Furthermore, in this perspective, modulating the oxidative balance appears as a new perspective of therapeutics for the treatment of chronic respiratory diseases, such as asthma, and new research is needed to better explore it.

## Figures and Tables

**Table 1:** Summary of main cells or effector systems altered in the airways due to increase of reactive species.

Cell or effector system	Effect
MAPK	Cell proliferation
Hydrogen peroxide	Smooth muscle contraction
8-Iso-PGF_2*α*_	Increase of airway resistance
3-Nitrotyrosine	Increase of airway inflammation
Neutrophil	Increase of airway inflammation
Nrf2/Keap1	Decrease of antioxidant activity
